# HIV integrase inhibitor, Elvitegravir, impairs RAG functions and inhibits V(D)J recombination

**DOI:** 10.1038/cddis.2017.237

**Published:** 2017-06-01

**Authors:** Mayilaadumveettil Nishana, Namrata M Nilavar, Rupa Kumari, Monica Pandey, Sathees C Raghavan

**Affiliations:** 1 Department of Biochemistry, Indian Institute of Science, Bangalore 560 012, India

## Abstract

Integrase inhibitors are a class of antiretroviral drugs used for the treatment of AIDS that target HIV integrase, an enzyme responsible for integration of viral cDNA into host genome. RAG1, a critical enzyme involved in V(D)J recombination exhibits structural similarity to HIV integrase. We find that two integrase inhibitors, Raltegravir and Elvitegravir, interfered with the physiological functions of RAGs such as binding, cleavage and hairpin formation at the recombination signal sequence (RSS), though the effect of Raltegravir was limited. Circular dichroism studies demonstrated a distinct change in the secondary structure of RAG1 central domain (RAG1 shares DDE motif amino acids with integrases), and when incubated with Elvitegravir, an equilibrium dissociation constant (*K*_d_) of 32.53±2.9 *μ*M was determined by Biolayer interferometry, leading to inhibition of its binding to DNA. Besides, using extrachromosomal assays, we show that Elvitegravir inhibited both coding and signal joint formation in pre-B cells. Importantly, treatment with Elvitegravir resulted in significant reduction of mature B lymphocytes in 70% of mice studied. Thus, our study suggests a potential risk associated with the use of Elvitegravir as an antiretroviral drug, considering the evolutionary and structural similarities between HIV integrase and RAGs.

Human immunodeficiency virus (HIV) infection leads to acquired immunodeficiency syndrome (AIDS). Reverse transcriptase, protease and integrase are the enzymes essential for the replication of HIV in host cells and hence all of these have been considered as drug targets against its infection. Integrase inhibitors have been recently developed as one of the most promising modalities for HIV therapy.^1^ Integrase is responsible for the insertion of HIV cDNA into the host genome. Before integration, integrase processes the 3′ ends of cDNA resulting in the exposure of a conserved CA sequence, yielding two reactive 3′ hydroxyl (OH) groups (Figure 1a). Following this, a pre-integration complex is formed which is transported to the nucleus, where the OH groups are utilised in a nucleophilic attack on the host genome leading to strand transfer.^2, 3^

Integrase is structurally similar to ribonucleases (RNases) and recombination activating gene 1 (RAG1), although they lack significant sequence similarity (Figure 1c and d). The RAG complex, comprising of RAG1 and RAG2, is essential for the generation of antigen receptor diversity and its absence leads to immunodeficiency diseases.^4, 5, 6, 7^ RAG1 and integrase share homology in the N-terminal zinc finger domain that aids in dimerisation, the central catalytic core domain, essential for enzymatic activity and the C-terminal domain that binds DNA non-specifically (Figure 1c and d).^3, 8^ The arrangement of secondary structure elements and the position of two catalytic aspartic acid residues relative to these elements are also comparable between RAG1 and integrase.^8, 9^ Besides, there is a striking functional similarity observed among the two proteins (Figure 1a and b). RAG complex binds to the recombination signal sequence (RSS) and cleaves precisely at its heptamer (CACAGTG). Similar to sequence-specific cleavage of RAGs, integrase cleaves exactly at the terminal dinucleotide of viral cDNA. Cleavage by RAGs is followed by a transesterification reaction, a mechanism shared by both transposases and retroviral integrases (Figure 1a and b).^2, 10, 11^ Both proteins catalyse disintegration reactions and mediate alcoholysis/hydrolysis during the nicking step. In the presence of Ca^2+^, integrase binds to DNA and forms protein–DNA complexes, which cannot catalyse cleavage reactions, a mechanism analogous to that of RAGs.^12^ RAG1 and integrase harbour a conserved catalytic DDE motif and the carboxyl groups of the amino acids harbouring this motif bind one or more divalent ions like Mg^2+^, which are essential for the activity of both the proteins.^10, 13^

An earlier study on two integrase inhibitors; 5CITEP^14^ and p10^15^ showed that they interfere with various physiological activities of RAG *in vitro*.^16^ Further development of drugs with CITEP as the lead compound was curtailed owing to its rapid clearance from the body by glucuronidation.^17^

Raltegravir (MK-0518), commercially known as Isentress (Merck & Co.), a structural analogue of the diketo acid class of compounds, is the first drug in the integrase inhibitor class approved by United States Food and Drug Administration (FDA) for the treatment of HIV-1^1^ (Figure 2a). Elvitegravir (GS-9137), a quinolone carboxylic acid strand-transfer-specific inhibitor by Gilead Sciences is also approved by FDA for therapeutic use (Figure 2b). Both these compounds share a *β*-hydroxy-ketone structural motif that possesses metal-chelating functions, and are believed to interact with divalent metals in the active site of the integrase.^18^

There are reports on early onset of autoimmune diseases following treatment with integrase inhibitors.^19^ An unexpectedly high rate of Non-Hodgkin’s lymphoma was also reported among patients undergoing treatment with integrase inhibitors.^20^ Taking the reports into account, National Institutes of Health had proposed a clinical trial with a hypothesis that ‘treatment with Raltegravir does not alter V(D)J recombination or immune responses to neoantigens’ (ClinicalTrials.gov Identifier: NCT00785967). However, the study was prematurely terminated because of difficulty in recruitment of volunteers (ClinicalTrials.gov Identifier: NCT00785967).

In the current study, we have tested two integrase inhibitors, Raltegravir and Elvitegravir, for their effects on the activity of RAG complex. Results showed that Elvitegravir inhibited various physiological activities of RAGs, while Raltegravir showed no significant effect. Therefore, our studies reveal a potential threat by Elvitegravir to the immune system during the course of its usage.

## Results

### Integrase inhibitors inhibit binding, cleavage and hairpin formation by RAGs at RSS

**Figure 1:**
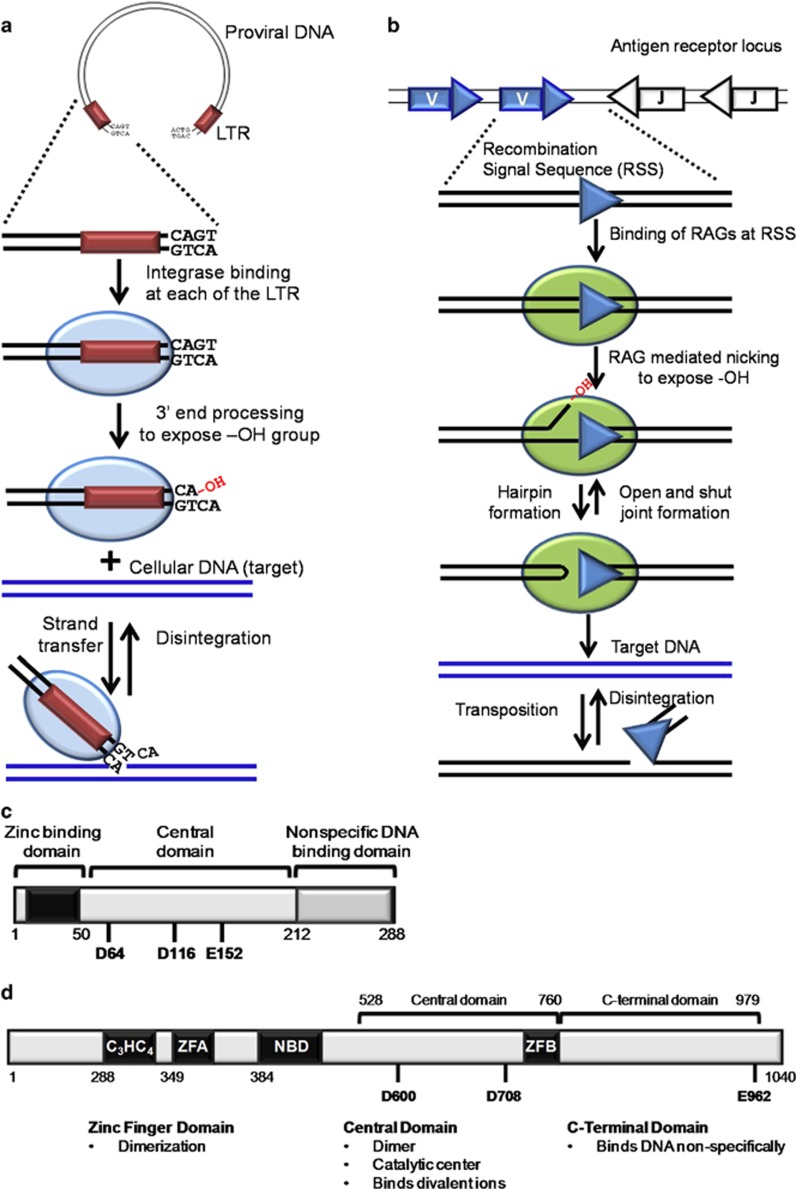
Similarities between HIV1 integrase and RAG1. (**a**) HIV integrase (blue oval) binds to the long terminal repeats/LTR (maroon box) near the ends of viral cDNA. It nicks the LTRs at a conserved ‘CA’, leaving a free 3′-OH group that can attack the cellular (target) DNA by a process called ‘strand transfer’. This process is reversible and is termed ‘disintegration’. (**b**) RAG complex (green oval) binds to RSS (white/blue triangles) and induces a nick 5′ to it. The 3′-OH group so created, attacks the opposite strand leading to formation of a hairpin at the coding end and a blunt signal end. The signal end can attack target DNA non-specifically by a process termed ‘transposition’. Both hairpin formation and transposition are reversible leading to ‘open-and-shut joint formation’ and ‘disintegration’, respectively. (**c**) Schematic representation of domains of HIV1 integrase. The zinc binding domain with its C_2_H_2_ motif (black box), catalytic core domain with DDE motif and the nonspecific DNA binding domain at C-terminal is shown. (**d**) Schematic representation of RAG1 domains. The zinc binding domains, central domain and C-terminal domain are shown. C_3_HC_4_ and Zinc Finger A (ZFA) forms the N-terminal dimerisation domain. Nonamer binding domain (NBD), Zinc finger B (ZFB) and the triad of catalytic amino acids, DDE is also marked. The functions of each domain conserved among RAG1 and HIV1 integrase are listed

**Figure 2:**
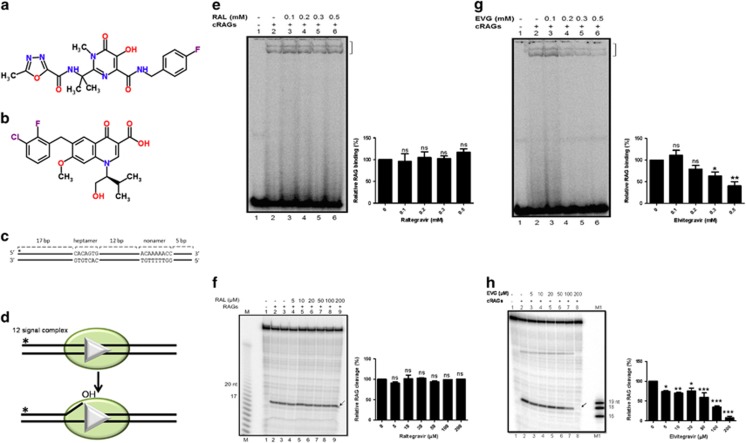
Effect of integrase inhibitors on physiological activity of RAGs. (**a** and **b**) Chemical structure of integrase inhibitors, Raltegravir (CSID:16445111) (**a**) and Elvitegravir (CSID:4441060) (**b**) used in the study. (**c**) Schematic representation of the oligomeric DNA substrate containing 12RSS used in the assay. The heptamer and nonamer are indicated and the asterisk indicates position of [γ-^32^P] end label. (**d**) Schematic representation of RAG binding and subsequent cleavage at standard RSS. The 12RSS is represented as white triangle and RAG proteins as shaded green oval. (**e**) Gel profile showing the effect of Raltegravir (RAL) on RAG binding at 12RSS. Following incubation of the DNA with RAGs and increasing concentrations of RAL (0, 0.1, 0.2, 0.3, 0.5 mM), the products were resolved on a 6% native polyacrylamide gel. The bands due to RAG binding are indicated by a square bracket; the lower band corresponds to RAG1 binding to DNA, while upper band corresponds to RAG complex binding. (**f**) Gel profile showing the effect of Raltegravir on RAG cleavage at 12RSS. DNA was subjected to RAG-mediated cleavage in a buffer containing 5 mM MgCl_2_ and increasing concentrations of RAL (0, 5, 10, 20, 50, 100, 200 *μ*M) and the products were resolved on a 15% denaturing PAGE. RAG cleavage products of 17 nt are indicated by arrow. M represents a single nucleotide ladder. (**g** and **h**). Effect of Elvitegravir (EVG) on RAG binding and cleavage at 12RSS. The concentrations of EVG used are same as above and indicated. M1 is molecular weight marker of 16, 18 and 19 nt. In **e**–**h**, bar diagram presented at the right of the gel shows the quantification of the RAG bound or cleaved products following treatment with inhibitors. Quantitation was performed with respect to the RAG bound or cleaved products in the control reactions and plotted as relative binding or cleavage in %. In each case, the data shown is obtained from three independent experiments

During V(D)J recombination, RAGs bind to RSS in the antigen receptor loci in a sequence-specific manner and cleave precisely at the 5′ end of the heptamer (Figure 1). Since RAG1 shares structural and functional similarity with HIV integrase (Figure 1), we tested whether Raltegravir and Elvitegravir, affect the sequence-specific action of RAGs (Figure 2a and b). For this, purified core RAG (cRAG) proteins were incubated with increasing concentrations of inhibitors (Figure 2c and d). Results showed no significant inhibition in binding and cleavage mediated by cRAGs at 12RSS when incubated along with Raltegravir (Figure 2e and f). However, Elvitegravir significantly inhibited both binding and cleavage, in a concentration dependent manner (Figure 2g and h). Although a distinct inhibition in RAG binding to RSS by Elvitegravir was seen from a concentration of 200 *μ*M onwards, inhibition of cleavage was observed at concentrations as low as 50 *μ*M (Figure 2g and h).

**Figure 3:**
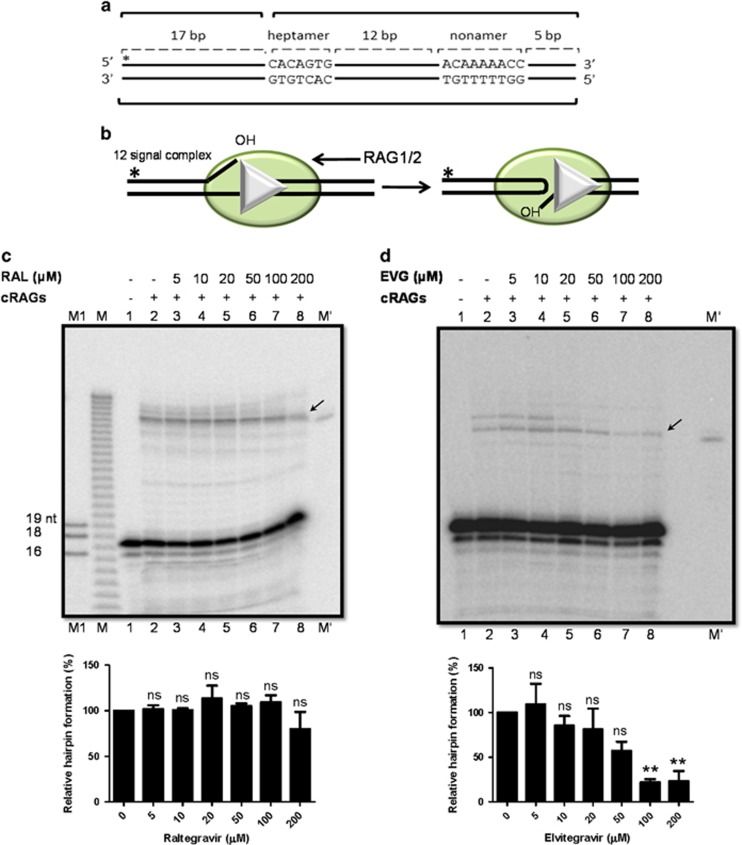
Effect of integrase inhibitors on hairpin formation during V(D)J recombination. (**a**) Schematic representation of the pre-nicked oligomeric DNA substrate used in the assay. (**b**) Schematic representation of RAG-mediated hairpin formation at RSS. (**c** and **d**) Gel profile showing the effect of Raltegravir or Elvitegravir on hairpin formation by RAGs. The labelled DNA was incubated with RAGs and increasing concentrations of inhibitors (0, 5, 10, 20, 50, 100 and 200 *μ*M) in a buffer containing 5 mM MnCl_2_ and the products were separated on a denaturing PAGE. Band due to hairpin formation is indicated by arrows. M represents a single nucleotide DNA ladder, M′ is a hairpin marker and M1 represents a molecular weight marker for 16, 18 and 19 nt. Relative quantifications of the RAG-mediated hairpin formation in the presence of inhibitors are shown below the gels. The data shown are the quantification from three independent batches of experiments

To determine whether integrase inhibitors can block hairpin formation during V(D)J recombination, pre-nicked 12RSS (Figure 3a and b) were incubated with RAGs in presence of increasing concentrations of inhibitors in a buffer containing 5 mM MnCl_2_ that supports hairpin formation. Raltegravir exhibited some degree of inhibition (Figure 3c), whereas Elvitegravir inhibited hairpin formation from 50 *μ*M onwards (Figure 3d). Thus, Elvitegravir inhibited the physiological activity of RAGs at different stages such as binding, nicking and hairpin formation, while Raltegravir showed no significant effect.

### The structure-specific cleavage mediated by RAGs is inhibited by integrase inhibitors

**Figure 4:**
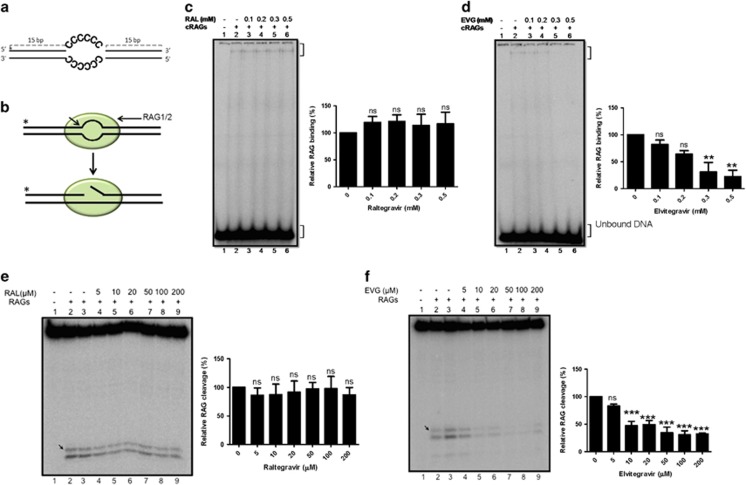
Effect of integrase inhibitors on structure-specific cleavage activity by RAGs. (**a**) Schematic representation of the oligomeric DNA substrate containing heteroduplex DNA (C/C)_6_ used in the assay. The double stranded DNA is shown in bold and the sequence of the region forming the bubble is indicated. (**b**) Schematic representation of RAG binding and cleavage at heteroduplex DNA. (**c** and **d**) Gel profile showing the effect of Raltegravir and Elvitegravir on RAG binding at heteroduplex DNA harbouring 6 nt cytosine bubble. (**e** and **f**) Gel profile showing the effect of inhibitors on RAG cleavage at heteroduplex DNA. RAG cleavage position is indicated by arrow. For other details refer figure legend 2. The relative quantifications of the RAG bound or cleaved products (derived from three independent batches of experiments) are shown towards the right in each panel

In addition to sequence-specific activity, the RAG complex also exhibits a pathological activity.^5, 21, 22^ Hence, we assessed the inhibition of RAG-mediated binding and cleavage using oligomeric DNA harbouring single-stranded cytosines as a (C/C)_6_ bubble, previously shown to have highest RAG cleavage^23^ with increasing concentrations of integrase inhibitors (Figure 4a and b). Elvitegravir inhibited RAG-mediated binding at (C/C)_6_ bubble from 100 *μ*M onwards and cleavage beginning at 10 *μ*M (Figure 4d and f), while Raltegravir showed no significant effect on either binding or cleavage (Figure 4c and e). Therefore, integrase inhibitors can impair both sequence and structure-specific activity of RAGs.

### Elvitegravir introduces structural changes in the central domain of RAG1 but not C-terminal domain

**Figure 5:**
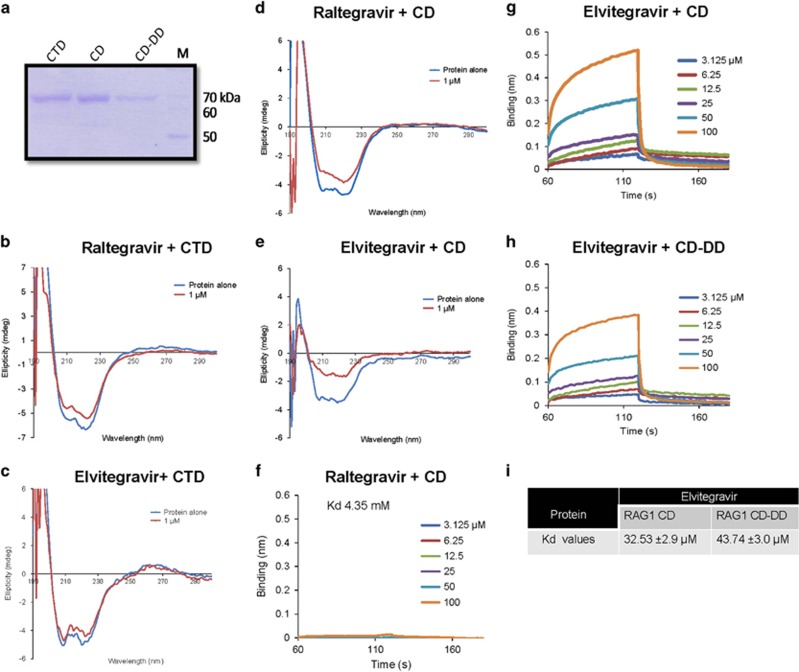
Circular dichroism spectroscopy and biolayer interferometry to evaluate structural changes in different domains of RAG1 upon binding to integrase inhibitors. (**a**) SDS-PAGE showing the purified domains of RAG1 namely, C-terminal domain (CTD), central domain (CD) and central domain with the active site amino acids mutated (CD-DD). ‘M’ is molecular weight ladder. (**b**–**e**) The domains of RAG1 were incubated in presence 1 *μ*M integrase inhibitors at 4 °C for 10 min and the spectra was taken using JASCO J-810 spectropolarimeter with a scan range of 200–260 nm. Circular dichroism spectra of C-terminal domain of RAG1 following incubation with 1 *μ*M Raltegravir (**b**) and Elvitegravir (**c**). Spectra of central domain of RAG1 following incubation with 1 *μ*M Raltegravir (**d**) and Elvitegravir (**e**). In all the cases, the spectrum of buffer with equivalent concentration of inhibitor was subtracted from that of protein with inhibitor. (**f**–**h**) Biolayer interferometry studies showing differential binding of Elvitegravir and Raltegravir to CD and mutant CD-DD. Biolayer interferometry sensorgrams depicting the real time binding of different concentrations of Elvitegravir and Raltegravir (0, 3.125, 6.25, 12.5, 25, 50 and 100 *μ*M) to biotin-tagged purified proteins (1.4 nmol), CD (~70 kDa) (**f** and **g**) and mutant CD-DD (~70 kDa) (**h**) immobilised to SSA sensors. The sensorgrams curves depict the association, followed by dissociation of increasing concentration of inhibitors to the sensors. The real time binding curves were used to compute equilibrium dissociation constant (*K*_d_) by globally fitting the rate equation for 1:1 kinetics to the data. The dissociation constant (*K*_d_) value of Elvitegravir and Raltegravir for central domain of RAG1 is 32.53±2.9 *μ*M and 4.35 mM, respectively. The dissociation constant (*K*_d_) value of Elvitegravir for mutant CD-DD RAG1 is 43.74±3.0 *μ*M. (**i**) Table shows *K*_d_ values obtained based on multiple batches of BLI for RAG1 CD and CD-DD with Elvitegravir

RAG1 is composed of several topologically independent domains like the zinc-binding dimerisation domain (265–380), nonamer binding domain (residues 384–460), central domain (CD; residues 528–760) and C-terminal domain (CTD; residues 761–980).^13, 24^ Central domain of RAG1 harbours the two aspartic acid residues (DD) of the catalytic motif DDE and Zinc finger B (ZFB). In order to evaluate the effect of inhibitors on these independent domains, the C-terminal and central domains of RAG1 were purified (Figure 5a) and incubated with Raltegravir or Elvitegravir to study any changes on their conformation upon inhibitor binding using circular dichroism (Figure 5b and e). The C-terminal domain of RAG1 showed no significant changes in its secondary structure upon incubation with either of the integrase inhibitors (Figure 5b and c). However, the central domain exhibited a profound change following incubation with Elvitegravir (Figure 5e). Interestingly, a small shift was observed when central domain was incubated with 1 *μ*M Raltegravir (Figure 5d).

### Biolayer interferometry studies reveal binding specificity of Elvitegravir to central domain of RAG1 at single molecule level

In order to further test the binding affinity and specificity of Elvitegravir and Raltegravir to the central domain of RAG1 at single molecule level, we performed biolayer interferometry (BLI) assays.^25, 26, 27, 28^

The central domain of RAG1 was biotinylated and immobilised as ligand on to super-streptavidin (SSA) sensors and incubated with Elvitegravir and Raltegravir. The binding of small-molecule inhibitor to the ligand on the biosensor increases the optical thickness at the biosensor tip, which results in a wavelength shift. This in turn is measured in real time as a shift in the interference pattern. Results showed a significant shift in the association curve upon addition of Elvitegravir to central domain of RAG1 (Figure 5g), while the effect was minimal in presence of Raltegravir (Figure 5f). The real time binding curves of both the inhibitors were used to estimate equilibrium dissociation constants (*K*_d_) by globally fitting the rate equation for 1:1 (association: dissociation) kinetics to the data. Interestingly, results showed robust binding of Elvitegravir with CD of RAG1 with a *K*_d_ 32.53±2.9 *μ*M (Figure 5g and i). Consistent with biochemical results, binding affinity of Raltegravir to CD of RAG1 was significantly low (*K*_d_ 4.35 mM; Figure 5f).

The catalytic site of RAG1 is composed of a triad of amino acids (Asp600, Asp708 and Glu962), which are conserved among bacterial transposons and HIV integrases.^9, 13^ Bioinformatics studies indicated that integrase inhibitors interact with the DDE motif of HIV integrase.^18^ Since CD spectra showed that the binding of Elvitegravir is specific to central domain of RAG1, we investigated whether this interaction is specific to the DDE motif. For this, central domain of RAG1 harbouring point mutations at Asp600 and Asp708 (CD-DD) was overexpressed in *E. coli*, purified and subjected to BLI analysis (Figure 5a and f-i). Elvitegravir exhibited a reduced binding affinity with CD-DD (*K*_d_ 43.73±3.0 *μ*M), compared to that of wild type protein (Figure 5h and i). Hence, our results confirm the specific binding of Elvitegravir to central domain of RAG1. These results, in conjunction with above studies, suggest that Elvitegravir interferes with the central domain of RAG1 and in turn impedes its activities.

### Elvitegravir affects the signal joint formation during V(D)J recombination

**Figure 6:**
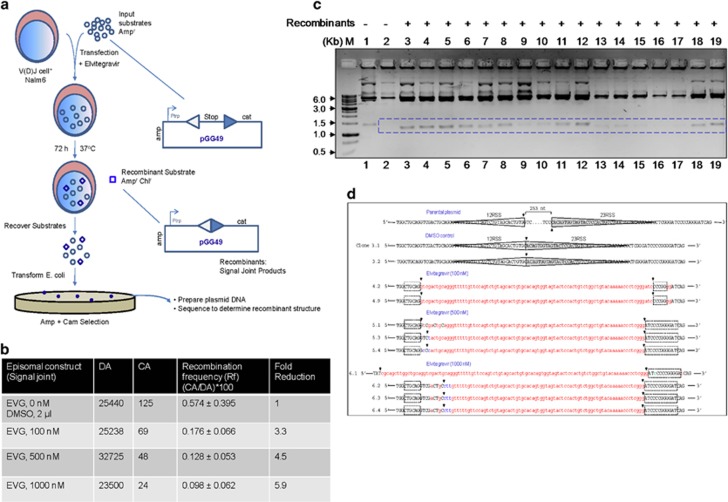
Evaluation of effect of Elvitegravir on V(D)J recombination using extrachromosomal assay system within pre-B cells. (**a**) Outline of the human V(D)J recombinase assay. The diagram depicts the introduction of plasmids into a human pre-B cell line, Nalm6, active for V(D)J recombination. After 72 h inside the cells (in presence and absence of Elvitegravir), the minichromosomes are harvested by Hirt harvest method and transformed into *E. coli* DH10B for detection of recombinants on LB agar plates containing ampicillin and chloramphenicol (Amp+Cam). The recombination is depicted between a consensus 12- (open triangle) and 23- (dark blue triangle) signal of pGG49, leading to signal joint formation. Recombination efficiency between 12RSS and 23RSS was analysed upon the treatment with different concentrations of Elvitegravir (0, 100, 500 and 1000 nM. ‘cat’ denotes the chloramphenicol acetyl transferase gene, and ‘stop’ denotes the prokaryotic transcription terminator. The *E. coli* trp promoter is denoted as ‘P_trp_’. The resistance gene for ampicillin is denoted as ‘amp’. Resistance for ampicillin and chloramphenicol is denoted as ‘amp^r^ cam^r^’. (**b**) Nalm6 cell line was transfected with pGG49 and the recombination efficiency was tested following transformation into *E. coli*. The recombination frequency (Rf) is calculated by the formula: (CA/DA) × 100, where the number of colonies obtained on ampicillin plate after DpnI digestion (DA) and chloramphenicol-ampicillin (CA)-selective media for the episomal substrate pGG49. Mean recombination frequency with standard deviation is presented. Fold change in recombination following treatment with different concentrations of Elvitegravir is also shown. (**c**) Agarose gel profile showing restriction digestion analysis of recombinants resulting from transfection of pGG49 in the presence of Elvitegravir. The bacterial colonies grown in double antibiotics (ampicillin-chloramphenicol) plates were inoculated in LB media. The plasmid DNA was isolated and double-digested with restriction enzymes, *Aat*II and *Bg*lII, to test for recombinants. Expected fragment release for recombinant plasmids is 1200 bp, while it is 1500 bp for a nonrecombinant DNA. ‘M’ is marker and dotted box indicates the band position for recombinants. Lane 1, is pGG49 digestion pattern (nonrecombinant). (**d**) DNA sequence analysis of recombinant junctions in pGG49. Parental plasmid represents the pGG49 sequence, which has not undergone recombination. The dotted line represents the sequence between 12 and 23RSS, which is 253 nucleotides in length. DMSO control indicates recombinants isolated from Nalm6 cells treated with vehicle control following transfection with pGG49. Sequence analysis showed precise signal joint formation between 12 and 23RSS after RAG-mediated recombination in vehicle control samples. Recombinant junctions derived following treatment with Elvitegravir (100, 500 and 1000 nM) in Nalm6 cells are also shown. 12 and 23RSS are highlighted using open and closed (grey) triangles, respectively. Extensive deletions, insertions and substitutions seen in these recombinants are indicated using red, green and blue colours, respectively. Black arrow shows the breakpoint region. Black dotted boxes represent palindromic sequences. Transfections were done a minimum of three independent times and multiple transformations were done using each batch

Biochemical studies suggest that Elvitegravir affects various properties of RAGs. Thus we evaluated its impact on V(D)J recombination in the RAG expressing pre-B-cell line, Nalm6, by employing an extrachromosomal assay.^29, 30, 31^ Cells were transfected with episomes harbouring the 12 and 23RSS (pGG49) in the presence of Elvitegravir.^30, 31, 32^ Recombination between two signals results in a signal joint formation (Figure 6a). Transfection products were transformed into *E.coli* to evaluate recombination frequency. Episomes harbour ampicillin (A) gene and gain chloramphenicol (CA) resistance as intervening transcription terminator is removed following recombination (Figure 6a). *DpnI* digestion was used to select plasmids that replicated inside mammalian cells, thereby providing replication frequency (DA). Recombination frequency (R) was deduced using the equation CA/DA × 100 (Figure 6b).

Results showed that number of CA colonies reduced upon treatment with Elvitegravir, although number of DA-resistant colonies remained high (Figure 6b). Thus, treatment with increasing concentration of Elvitegravir reduced the efficiency of recombination by 3–6-fold, as compared to vehicle or vector controls (Figure 6b). These results suggest that Elvitegravir affects V(D)J recombination inside human cells.

Plasmid DNA was isolated from recombinants obtained on CA plates to confirm their identity. Screening of recombinants by restriction enzyme digestion and agarose gel electrophoresis showed a band of ~1.5 kb in case of unrecombined original vector. A characteristic fragment of ~1.2 kb was seen in case of recombinants due to removal of the intervening sequence between 12 and 23RSS, leading to formation of signal joint (Figure 6a and c) that was confirmed by DNA sequencing as reported previously^31, 32^ (Figure 6d). Interestingly, in addition to the reduced recombination frequency, extensive deletions were observed when recombinants from Elvitegravir-treated samples were analysed. To our surprise, we did not find any recombinants that showed typical signal end joining of 12 and 23RSS in case of Elvitegravir-treated samples (Figure 6d). Furthermore, we observed breaks at two inverted repeats on each side of the junction in many Elvitegravir-treated clones. The relevance of this observation needs to be investigated further.

### Treatment with Elvitegravir reduces coding joint formation during V(D)J recombination

**Figure 7:**
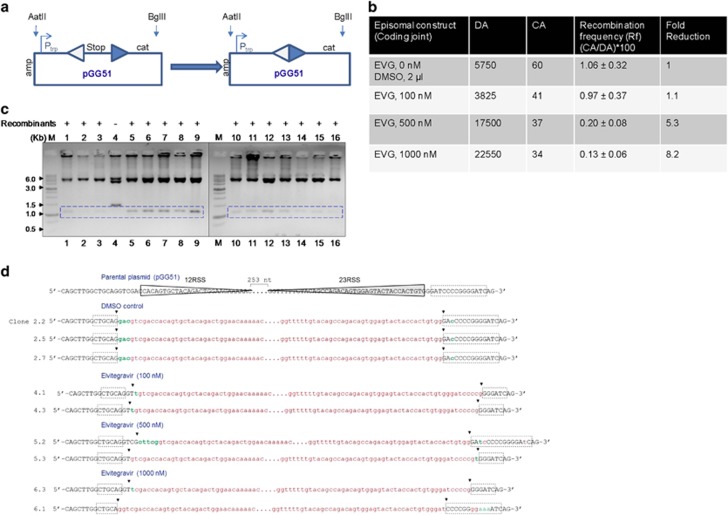
Impact of Elvitegravir on coding joint formation during V(D)J recombination. (**a**) The recombination is depicted between a consensus 12- and 23-signal of pGG51, leading to coding joint formation. (**b**) The episome pGG51 was transfected into the Nalm6, and the recombination efficiency in the presence of various concentrations of Elvitegravir was tested following transformation. (**c**) Restriction digestion analysis to check the presence of recombinants after transfection of pGG51 in Nalm6 in the presence of Elvitegravir. The plasmid DNA was isolated and double-digested with *AatII* and *BglII*. ‘M’ represents the marker and box indicates the band position for recombinants. (**d**) DNA sequence analysis of recombinant junctions in pGG51. For other details refer the legend of Figure 6

Nalm6 cells were transfected with an episome harbouring 12 and 23RSS (pGG51) such that coding joint formation can be studied, in the presence of increasing concentration of Elvitegravir (Figure 7a). Upon recombination between the 12 and 23RSS, transcription terminator will be excised out and the plasmid DNA will become resistant to ampicillin-chloramphenicol as described above (Figure 7a). Results showed upto eightfold reduction in recombination frequency in a concentration dependent manner, suggesting a significant inhibition in coding joint formation (Figure 7b). Evaluation of the CA-resistant colonies by restriction digestion confirmed the identity of the recombinants with a release of ~1.2 kb fragment (Figure 7c). Sequence analyses further confirmed the recombination between coding ends in case of episomal vector alone or vehicle control treated cells (Figure 7d). However, deletions of varying length were evident in Elvitegravir-treated samples (Figure 7d). Besides, there were insertions and substitutions in certain clones following Elvitegravir treatment (Figure 7d). Thus, our results suggest that treatment with Elvitegravir interfered with both coding and signal joint formation during V(D)J recombination. This further indicates that Elvitegravir treatment interferes with the physiological function of RAGs in human cells.

### Elvitegravir affects B cell development in mice

**Figure 8:**
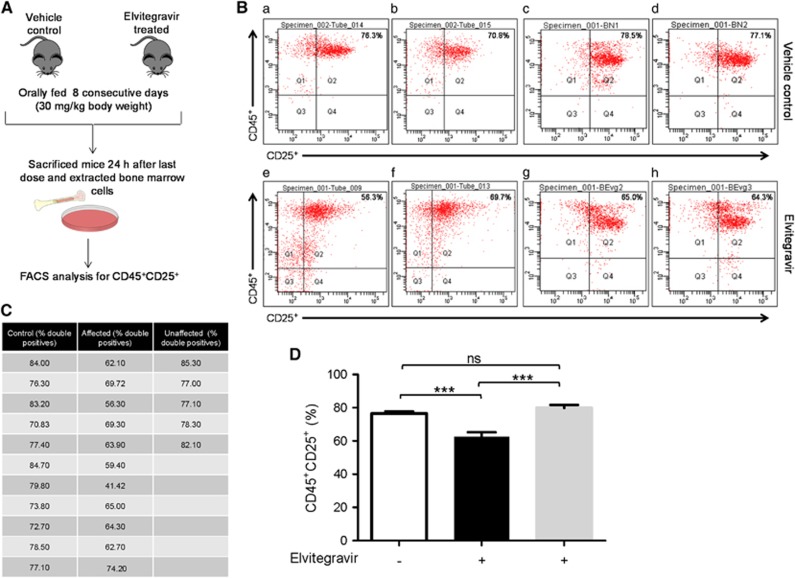
Evaluation of effect of Elvitegravir on B cells progression in mice by FACS analysis. (**A**) Schematic representation of steps involved during the *in vivo* experiment. Balb/c mice (vehicle control and Elvitegravir-treated) were fed with Elvitegravir (8 days; 30 mg/kg). Mice were sacrificed and bone marrow cells were collected, stained with CD45, CD25 surface markers and FACS analysed. (**B**) Representative FACS dot plots of CD45^+^CD25^+^ cells from vehicle control and Elvitegravir-treated mice are shown. Two mice each from independent batches of vehicle control (a,b batch I, c,d batch II) and Elvitegravir-treated groups (e,f batch I, g,h batch II) are presented. (**C**) Table showing percentage of CD45^+^CD25^+^ cells obtained following flow cytometric analysis from control (*n*=11) and Elvitegravir-treated (*n*=16) mice from all three batches. Mice, from the Elvitegarvir treated group, that are affected (*n*=11) and unaffected (*n*=5) by Elvitegravir are shown. (**D**) Histogram showing CD45^+^CD25^+^ B cells. Double-positive B cells detected following FACS analysis from bone marrow cells of vehicle control (white) are depicted in comparison to Elvitegravir-treated group which is divided into affected (black) and unaffected (grey). While vehicle control mice possessed average 78.03% of CD45^+^CD25^+^ B cells in bone marrow it was reduced to average 62.5% following Elvitegravir treatment. ~30% mice were insensitive to treatment. *P* value <0.001

To understand the effect of Elvitegravir on the immune system, lymphocytes from the bone marrow were analysed from 2–3 weeks old Balb/c mice, which were treated with Elvitegravir and its stabilising agent, Cobicistat (Cobicistat is a cytochrome P450 inhibitor, which helps in the stabilisation of Elvitegravir by inhibiting its degradation).^33^ The compounds were orally fed to mice for a period of eight consecutive days followed by analysis of bone marrow cells (Figure 8a). Cells were analysed via FACS using specific cell surface markers, CD45 and CD25, to understand the effect of Elvitegravir on B-cell development *in vivo,* after gating the total lymphocyte population. CD45 is constitutively expressed in B and T cells, while CD25 is expressed in the activated B and T cells.^34, 35, 36, 37^ In order to investigate the effect of Elvitegravir, we evaluated difference in CD45^+^CD25^+^ B cell population, where a decrease in double-positive cells would implicate the role of Elvitegravir in affecting RAG activity during pro and pre-B-cell stages leading to a reduction in mature CD45^+^CD25^+^ B cells.

In order to assess the percentage of B and T cells in the bone marrow, cells were flushed out from mice bone marrow (*n*=6) and stained with CD3^+^CD4^+^CD8^+^ (T cell specific surface markers)^38, 39^ or with CD19^+^CD25^+^ surface markers.^40, 41^ CD19 is a pan B cell marker. The majority of lymphocytes in the bone marrow cells were found to be B cells (86–90%) and, T-cells were approximately 4–5%, suggesting that the double-positive cells (CD45^+^CD25^+^) mostly consist of B cells (data not shown).

A total of 16 mice were treated with Elvitegravir and Cobicistat, (30 mg/kg body weight) and 11 mice were treated with vehicle control (methylcellulose) distributed across three independent batches. FACS analyses of B cell population revealed ~78% CD45^+^CD25^+^ cells in the case of mice treated with vehicle control, whereas this population was significantly reduced in 11 of the 16 Elvitegravir-treated mice (Figure 8b–d). Interestingly, 5 out of 16 mice did not show any significant difference in CD45^+^CD25^+^ cells, compared to vehicle control (Figure 8b–d), and this group of mice were reported as ‘unaffected’ (Figure 8c and d). We also tested a group of four mice to study the impact of Cobicistat (30 mg/kg b.wt) treatment on their CD45^+^CD25^+^ cells. Flow cytometry studies after isolating bone marrow cells suggested that there was no significant difference in CD45^+^CD25^+^ positive cells in this population (data not shown). Therefore, our results reveal that there is a significant decrease in the mature B-cell population upon treatment with Elvitegravir in ~70% of the animals, although ~30% of the animals were unaffected. These results, in conjunction with *in vitro* biochemical and *ex vivo* studies, suggest that Elvitegravir could interfere with RAG function during B-cell development leading to failure of V(D)J rearrangement in pre-B cells, which in turn results in reduced levels of mature B cells.

## Discussion

### Elvitegravir inhibits various stages of V(D)J recombination

RAG complex is an essential enzyme that aids in the development of immunity in mammals. However, it shares a common active site with HIV integrase. The present study shows that among the integrase inhibitors currently available in the market, Elvitegravir, and not significantly, Raltegravir, inhibited the biochemical functions of RAGs. Specifically, it inhibited binding and cleavage at RSS, and hairpin formation. RAG cleavage at RSS was inhibited at a lower concentration of Elvitegravir compared to that of its binding. In addition to its effect on sequence-specific cleavage at RSS, Elvitegravir inhibited the structure-specific activity of RAGs. Interestingly, the second integrase inhibitor studied, Raltegravir, exhibited only limited inhibition.

By using *ex vivo* episomal assays, we observed that Elvitegravir affected V(D)J recombination in Nalm6 cells in a concentration dependent manner by affecting joining of both coding and signal ends. Recombination frequency was reduced upto 6-fold when signal joints were analysed, while it was reduced upto 8-fold during coding joint formation. This is understandable as the efficiency of formation of signal joint is higher compared to coding joint during V(D)J recombination within cells.^42^ Further, sequence analysis of the recombinant junctions revealed that Elvitegravir treatment resulted in junctional sequence alterations in both coding and signal joints, and was different when compared to untreated controls. Besides the observed lower recombination frequency, Elvitegravir-treated samples also exhibited extensive deletions. In fact, sequencing of recombinants resulting from Elvitegravir treatment suggested that RAG cleavage occurred at certain palindromic sequences present upstream and downstream of RSS rather than at the expected 5′ end of the heptamer. Alternatively, the junctions could have been processed by a nuclease following RAG cleavage in a minor fraction of DNA molecules. The joining at the palindromic sequences is suggestive of the use of alternative-NHEJ over classical-NHEJ for repair of DNA breaks; however, this aspect needs further investigation.

### Integrase inhibitors act against the common active site shared between RAGs and integrase

Molecular docking studies have shown that Raltegravir and Elvitegravir make direct interactions with the amino acids of the DDE motif in the integrase.^18^ In addition, another inhibitor of the diketo acid class, 5CITEP, has been shown to act by sequestering metal ions essential for integrase action. In the currently accepted model of inhibitor action, the two metal ions bound at the DDE motif are coordinated by the inhibitor.^43^ Furthermore, several mutations reported in patients like T66I, S153Y and M154I, that confer resistance to integrase inhibitors are positioned adjacent to the amino acids of DDE motif, namely, D64, D116 and E152.^15, 44^ These studies suggest that the integrase inhibitors interact with DDE motif of integrase. Interestingly, circular dichroism studies using central domain of RAG1, which possesses two aspartates of the DDE motif, indicated profound changes in its secondary structure upon incubation with Elvitegravir. The inhibition of binding of central domain to 12RSS containing DNA substrates in presence of Elvitegravir was specific with a *K*_d_ value of 32.53±2.9 *μ*M, as determined using BLI, further confirming its specificity towards central domain of the protein. Several studies, including crystallography, on the retroviral integrases suggest that aspartates are involved in the binding of a single Mg^2+^.^45, 46, 47, 48^ Interestingly, we observe that the central domain of RAG1 that lacks the glutamate of the DDE motif was also inhibited by Elvitegravir.

### Implications of the effect of integrase inhibitors on RAGs

**Figure 9:**
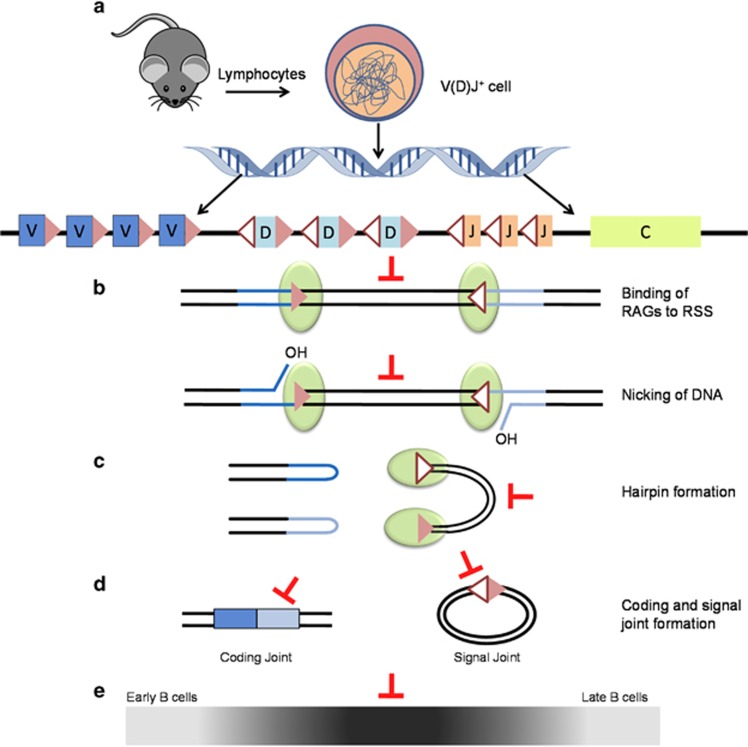
Model depicting potential impact of Elvitegravir during various stages of V(D)J recombination within B cells. (**a–e**). Lymphocytes were obtained from Balb/c mice or from cell lines. Cells positive for V(D)J recombination were analysed by FACS (*in vivo*) or extrachromosomal assay (*ex vivo*) to understand the effect of Elvitegravir on RAG function. During the process of V(D)J recombination, RAGs bind to the RSS (**a** and **b**) and introduces a nick at the 5′ end of the heptamer (**b**). The exposed OH group attacks the opposite strand by the process of transesterification leading to the creation of hairpin tipped coding ends and blunt ended signal ends (**c**). Coding ends further undergo processing by NHEJ, while the signal ends are joined without processing (**d**). Different subexons in the IgH locus are indicated using different colours viz*.* dark blue for V segments; light blue depicts D segments; orange for J segments and yellow highlights the constant region of the V(D)J locus (**a**). Green ovals represent the RAG proteins (RAG1 and RAG2) (**b**). Open triangles show 12RSS whereas, closed triangles represent the 23RSS. Coding and signal joints are depicted as joined products of V and D segments, and 12RSS and 23RSS, respectively. The gradient depicts the maturation of B cells. The stages of V(D)J recombination blocked due to treatment with Elvitegravir are indicated using red blunt headed arrows. Elvitegravir inhibits RAG-mediated cleavage and hairpin formation at a lower concentration, while the binding is inhibited only at high concentration of the inhibitor, based on *in vitro* RAG cleavage assay

Our results show that Elvitegravir can have an impact on lymphocyte maturation, particularly B-cell maturation in ~70% of the mice tested, when a dose equivalent to that administered in human patients was used. We observed significant reduction in CD45^+^CD25^+^ double-positive cell population following FACS analysis of B lymphocytes. As stated above, the effect of Elvitegravir on RAG functions is to inhibit the progression of pro B/pre-B cells to the next stage, which in turn could lead to a decline in the mature cell population (Figure 9). Since some mice were resistant to such a change, it appears that every organism may not be equally susceptible to Elvitegravir. However, the observed results suggest that treating patients with Elvitegravir can have an adverse secondary impact on their immune system.

Since we do not see a huge decrease in the B and T cell numbers, even in affected mice, it is possible that other factors such as efficiency of Cobicistat in stabilising Elvitegravir, doses used, and the treatment regime selected in the present study (eight days) affect the outcome. In humans infected with HIV, the treatment regime is daily one tablet over a lifetime. Although our results showed the effect of inhibitors is limited, the impact in humans can be significantly higher since the drug administration is over a long period of time. However, the impact of Elvitegravir on V(D)J recombination in patients using integrase inhibitors needs to be tested more comprehensively.

## Materials and Methods

### Enzymes, chemicals and reagents

Chemicals and reagents used in the study were purchased from Sigma Chemical Co. (St. Louis, MO, USA), Amresco (Solon, OH, USA) and SRL (India). Restriction enzymes and other DNA-modifying enzymes were obtained from New England Biolabs (Ipswich, MA, USA). Culture media were from Sera Laboratory International Limited (West Sussex, UK). Foetal bovine serum and PenStrep were from Gibco BRL (Carlsbad, CA, USA). Radioisotope-labelled nucleotides were from BRIT (Hyderabad, AP, India).

Integrase Inhibitors, Raltegravir (MK-0518) and Elvitegravir (GS-9137) were purchased from Selleck Chemicals (Houston, TX, USA). Cobicistat (1004316-88-4) was purchased from Shanghai Sun-shine Chemical Technology Co. Ltd., (Wuhan, China).

### Antibodies

Antibodies were purchased from Santa Cruz Biotechnology (USA), BD Biosciences (USA) and Imgenex (Bhubaneswar, Odisha, India). Anti-MBP antibody (Cat No. 808) was from Santa Cruz. Anti-CD3-FITC (Cat No. 555274), anti-CD8-APC-Cy7 (Cat No. 557654), anti-CD19-APC (Cat No. 550992), anti-CD25-PECy7 (Cat No. 552880) and anti-CD45-APC (Cat No. 559864) were from BD Biosciences. Anti-CD4-PE (Cat No. 5922D) was purchased from Imgenex.

### Plasmids

Plasmids encoding MBP-tagged murine cRAGs and HMGB1 were gifted by Dr. P. Swanson, USA. pGG49 and pGG51 were from Dr. M. Lieber, USA. Plasmids pRS3 and pJLA1 encoding domains of RAG1, namely, central domain (amino acids 528–760) and C-terminal domain (761–980) as well as the construct for central domain with point mutations D600A/D708A were kind gifts from Dr. K. Rodgers, USA.

### Cell lines and culture

Human embryonic kidney cell line expressing Simian Virus 40 large tumour (T) antigen, 293 T was grown in DMEM high glucose with L-glutamine containing 10% FBS. Pre-B cell line, Nalm6 was grown in RPMI medium containing 10% FBS. The cells growing in log phase were used for transfection.^29^ Medium was supplemented with 100 *μ*g/ml Penicillin G and streptomycin, and incubated at 37 °C in a humidified atmosphere containing 5% CO_2_.

### Oligomeric DNA

Oligomers used in the study were synthesised from Sigma-Aldrich (Bangalore, KA, India). The oligomers were purified using 10–15% denaturing PAGE, as described.^49^

### Preparation of oligonucleotide dsDNA substrates

The oligomeric DNA containing 12 and 23RSS were created by annealing [γ-^32^P] end-labelled oligomers, AKN1: (5′-GATCAGCTGATAGCTACCACAGTGCTACAGACTGGAA-CAAAAACCCTGCT-3′) or AKN3 (5′-GATCAGCTGACAGTAGCACAGTGGTA-GTACTCCACTCTCTGGCTGTACAAAAACCCTGCT-3′) with unlabelled complementary oligomers AKN2 (5′-TAGCAGGGTTTTTGTTCCAGTCTGTAGCACTGTGGTAGCTATCAGCTGAT-3′) or AKN4 (5′-TAGCAGGGTTTTTGTACAGCCAGAGAGTGGAGTACTACCACTGTGCTACTGTC-AGCTGAT-3′), respectively.^23^ Labelled AKN46 (5′-GACCTGAGGGCGAGCCCCCCCCGAGTAACTTAACAG-3′) was paired with unlabelled AKN20 (5′-CTGTTAAGTTACTCGCCCCCCGCTCGCCCTCAGGTC-3′) to generate a heteroduplex DNA harbouring a six-nucleotide CCCCCC/CCCCCC as bubble sequence.^23^ Oligomeric DNA corresponding to pre-nicked RSS was prepared by annealing labelled MN50 (5′-GATCAGCTGATAGCTAC-3′) with unlabelled MN51 (5′-CACAGTGCTACAGACT-GGAACAAAAACCCTGCT-3′) and AKN2.^29^ MN65 (5′-GATCAGCTGATAGCTACGTAGCTATCAGCTGATC-3′) was used as a marker for hairpin DNA. Primers SCR105 (5′-GGCGTATCACGAGGCCCTTTCGTC-3′) and SCR 190 (5′-TAGGTACATTGAGCAACTGAC-3′) were used for the sequencing of recombinants in the episomal assays.

### 5′ end-labelling of oligomers

5′ end-labelling of oligomeric DNA was performed using T4 polynucleotide kinase and [γ-^32^P] ATP at 37 °C for 1 h as described.^50^ Labelled substrates were purified using Sephadex G-25 column and stored at −20 °C till use. Radiolabelled duplex DNA was prepared by annealing labelled strand with fivefold excess of complementary unlabelled strand in 100 mM NaCl and 1 mM EDTA in a boiling water bath, followed by gradual cooling, as described earlier.^51, 52^

### Expression and purification of proteins

The mammalian expression constructs, pcDNA1 harbouring core RAG1 (cRAG1, amino acids 384–1040) and core RAG2 (cRAG2, amino acids 1–383) each fused at its N-terminus to the maltose binding protein (MBP) was used for expression.^41^ The protein was purified as described previously.^53, 54, 55^ In brief, HEK 293 T cells were transfected with plasmid constructs using Calcium phosphate method. Cells were harvested after 48 h of transfection, protein lysate was prepared and RAG proteins were purified using amylose resin column chromatography. The central and C-terminal domains of RAG1 as well as central domain with D600A/D708A mutations were expressed in *Escherichia coli* BL21 (DE3) cells as described earlier.^56^ HMGB1 was purified by virtue of its histidine tag following expression in *E. coli* BL21 (DE3). In all cases the proteins were judged to be 95% pure by coomassie blue staining of SDS-PAGE gels and identity was confirmed by western blotting.

### Electrophoretic mobility shift assay

Electrophoretic mobility shift assay was carried out as described previously.^53, 54, 57^ In brief, appropriate [γ-^32^P] ATP-labelled oligomeric DNA substrates were incubated with RAG proteins (100 ng) in a buffer containing 22.5 mM MOPS-KOH (pH 7.0), 20% DMSO, 2.2 mM DTT, 50 mM potassium glutamate, 2% (v/v) glycerol and 100 ng/ml BSA for 2 h at 25 °C following which fixation was carried out (0.01% v/v gluteraldehyde at 37 °C for 10 min). The DNA–protein complexes were allowed to form in the presence of 5 mM CaCl_2_ and increasing concentration of integrase inhibitors (0, 0.1, 0.2, 0.3 and 0.5 mM) and resolved on native polyacrylamide gels (6%). The gels were dried and signals detected using a PhosphorImager, FLA9000 (Fuji, Japan). Each experiment was done a minimum of three independent times with complete agreement.

### RAG cleavage assay

RAG cleavage assay was performed in a buffer containing 25 mM MOPS (pH 7.0), 30 mM KCl, 30 mM potassium glutamate and 5 mM MgCl_2_ as described.^23, 55, 58^ Radiolabelled oligomers and RAGs were incubated at 37 °C for 1 h in presence of Raltegravir or Elvitegravir (0, 5, 10, 20, 50, 100 and 200 *μ*M) dissolved in DMSO. In control reactions, RAG reaction buffer and equivalent volume of DMSO was used. Reactions were terminated by adding loading dye containing formamide, heated for 10 min at 95 °C and resolved on 15% denaturing polyacrylamide gels. The gels were dried and images were acquired using a PhosphorImager. Each experiment described in the present study was done a minimum of three independent times with complete agreement. Cleavage products were quantified using Multi Gauge V3.0 software (Fuji Pharma, Tokyo, Japan). The relative cleavage was calculated and indicated.

### Hairpin formation by RAGs

To determine the effect of inhibitors on the hairpin formation by RAGs, increasing concentration of the inhibitors (0, 5, 10, 20, 50, 100 and 200 *μ*M) were incubated with labelled pre-nicked RSS, HMGB1 (facilitates hairpin formation) and RAGs.^29^ The reaction was done in a buffer containing 5 mM MnCl_2_ for 1 h at 37 °C and the products were separated on 15% denaturing PAGE. The gels were dried and images were acquired using a PhosphorImager (GE Healthcare, Chicago, IL, USA).

### Western blotting

For immunoblot analysis of RAGs, protein samples were resolved on 8% SDS-PAGE as described.^50, 59^ Following electrophoresis, the protein was transferred to PVDF membrane (Millipore, Billerica, MA, USA) blocked with 5% skimmed milk powder, probed with primary antibody against MBP and appropriate biotinylated secondary antibodies. The blots were developed using chemiluminscent solution (Immobilon western, Millipore) and scanned by gel documentation system (LAS 3000, Fuji, Japan).

### Circular dichroism spectroscopy

For circular dichroism studies, central domain (wild type) and C-terminal domain of RAG1 were overexpressed, purified and used. The protein was resuspended in phosphate buffered saline (PBS) and the spectrum was recorded at a wavelength of 200–260 nm at 4 °C using a JASCO J-810 spectropolarimeter as described.^51, 59^ 10 cycles were acquired for each sample at a scan speed of 100 nm per min. Integrase inhibitors were added to the purified proteins in PBS, incubated for 10 min on ice and the spectrum was recorded. For inhibitor studies, 1 *μ*M of Elvitegravir and Raltegravir were used. Spectra were also recorded for buffer alone and buffer containing corresponding concentration of DMSO or inhibitors that were subtracted from experimental data. The molar ellipticity was calculated using the software, Spectra Manager and plotted as a function of wavelength.

### Biolayer interferometry (BLI)

Central domain of RAG1 (CD) (wild type) and that with mutations D600A/D708A (CD-DD) were biotinylated (10 mg/ml) by incubating at room temperature for 1 h. The excess of biotin was removed using PD-25 size exclusion columns. ForteBio Octet RED 96 (Forte Bio, USA) and Super-Streptavidin (SSA) sensors (Forte Bio, USA) were employed for studying the binding of Elvitegravir and Raltegravir (analytes) to biotin-tagged proteins (ligand) immobilised onto the (SSA) sensors as described previously.^26, 27, 28^ 1 × PBS was used as the assay buffer for making serial dilutions of inhibitors and proteins, and the study was conducted at 30 °C. Before use, all the SSA sensor tips were hydrated in buffer (1 × PBS) for 10 min. 96-microwell plate filled with 200 *μ*l of buffer (1X PBS), containing either the inhibitor(s) or the equivalent DMSO in each case was agitated at 1500 rpm. To prevent nonspecific binding of protein to the SSA sensors, biocytin (1 mg/ml) was also included in the assay before exposing sensors to inhibitors. The programme was set up in a sequence of steps, which included establishment of a stable baseline with buffer (1 min), loading of sensors with biotin-tagged proteins, CD (Wild type) and CD-DD (5 *μ*g/ml, each) (6 min). A reference sensor without bound ligand (protein) was subjected to the same procedure as the sample sensors loaded with ligand, and then used for subtraction of the background signals during analysis. Following immobilisation of the two biotin-tagged proteins (1.4 nmol of CD RAG1 and 1.4 nmol CD-DD RAG1) on sensors, independent binding experiments with varying concentrations of Elvitegravir and Raltegravir (0, 3.125, 6.25, 12.5, 25, 50 and 100 *μ*M) were carried out, which included baseline (1 min), association (1 min), dissociation (1 min) steps corresponding to binding to each concentration of inhibitor, terminating the assay with baseline (8 min) and then *K*_d_ values were calculated by globally fitting the rate equation for 1:1 kinetics (association: dissociation) to the data, followed by their calculation for the proteins from the ratio of *k*_off_ (dissociation) and *k*_on_ (association) constants model for both the proteins towards Elvitegravir and Raltegravir.

### *In vivo* recombination assay

The human lymphoid cell line, Nalm6 was cultured and transfected with episomal constructs pGG51 (coding joint) or pGG49 (signal joint) along with increasing concentration of Elvitegravir (0, 100, 500 and 1000 nM) by electroporation (300 V, 900 *μ*F, ∞ Ω),^29^ and incubated for 72 h at 37 °C. Equivalent amount of DMSO served as vehicle control. Elvitegravir doses were selected on the basis of IC_50_ value of Nalm6, which was 8.9 *μ*M (data not shown). Thus, we selected lower concentrations to avoid toxicity of the compound. The DNA was recovered using Hirt harvest method^60^ and used for transforming *E. coli* DH10B. The transformation mixture was plated on ampicillin (A) and chloramphenicol-ampicillin (CA) LB agar plates. In brief, recombinant substrates will confer resistance to both the antibiotics. The ratio of CA colonies to A colonies reflects the fraction of recovered substrates that underwent V(D)J recombination. A more meaningful ratio can be obtained by focusing on those ampicillin-resistant molecules that have replicated inside the eukaryotic cells. *DpnI* digestion was performed on DNA substrates recovered following transfection (0.2 U, 37 °C for 1 h) to evaluate the replication status based on methylation pattern. The *DpnI* digested episomal DNA was transformed into *E. coli* DH10B, transformation mixture was plated on ampicillin containing LB agar plate, and DNA replicated inside the cells were designated as (DA). The actual recombination frequency was calculated using the formula (CA/DA × 100).

### Episomal DNA isolation from mammalian cells using Hirt harvest method

Mammalian cells were collected after 72 h of transfection with episomal constructs (pGG49, signal joint or pGG51, coding joint) as described previously.^29, 61^ Cells were spun down (1100 r.p.m. for 10 min at 4 °C), pellet was washed with PBS and resuspended in Hirt buffer (0.01 M Tris (pH 7.9), 0.01 M EDTA) and SDS (0.06%). 1/4th volume of 5 M NaCl was added and mixed by inverting the tubes. Following cell lysis, solution was incubated at 4 °C (overnight) and supernatant was collected by centrifugation (14 000 r.p.m., 30 min at 4 °C). Proteinase K (100 *μ*g/ml) was added to the supernatant (55 °C for 1 h) and DNA was extracted by phenol: chloroform (1:1) extraction and ethanol precipitation in presence of 1/10th volume of 3 M sodium acetate at −20 °C for >30 min. Centrifugation was done at 14 000 r.p.m. (30 min at 4 °C), supernatant was decanted carefully, and pellet was dried and resuspended in TE.

### DpnI digestion

To calculate the recombination frequency inside the cell, the plasmid isolated from mammalian cells was subjected to *DpnI* digestion in NEB CutSmart buffer (50 mM Potassium acetate, 20 mM Tris-acetate, 10 mM Magnesium acetate and 100 *μ*g/ml BSA, pH 7.9). 0.2 Unit of *DpnI* was taken per reaction in a total volume 10 *μ*l, incubated at 37 °C for 1 h. 1 *μ*l of *DpnI* digested plasmid DNA was transformed into *E. coli* DH10B, followed by incubation at 37 °C (for 1 h). The transformed culture was plated on LB agar plate containing ampicillin (100 *μ*g/ml). On the following day colonies were counted and replication frequency was calculated using the formula (DA/A) × 100, where A is the number of colonies obtained in Amp containing LB agar plate and DA is the number colonies in Amp plate after *DpnI* digestion.

### Restriction digestion and sequencing of recombinants

To confirm the process of recombination inside the cells, episomal DNA was isolated from the Ampicillin-Chloramphenicol (CA) resistant colonies using alkaline lysis method, followed by sequential double-digestion using the restriction enzymes *AatII* in NEB buffer 4 (50 mM Potassium acetate, 20 mM Tris-acetate, 10 mM Magnesium acetate and 1 mM DTT, pH 7.9) and *BglII* in NEB buffer 3 (100 mM NaCl, 50 mM Tris-HCl, 10 mM MgCl_2_, 1 mM DTT and pH 7.9) at 37 °C (4 h). Restriction digested products were resolved on agarose gel (1%) and images were captured using Gel Doc (LSA 3000). Positive clones were sequenced (Amnion Biosceinces, Bangalore, KA, India) and analysed.

### Balb/c mouse maintenance

#### Ethical statement

Mice were maintained as per the principles and guidelines of the ethical committee for animal care, Indian Institute of Science in accordance with Indian National Law on animal care and use. The experimental design of the present study was approved by Institutional Animal Ethics Committee (Ref. CAF/Ethics/366/2014), Indian Institute of Science, Bangalore, India.

Balb/c mice, 2–3 weeks old and weight 5-6 g were purchased from the central animal facility of Indian Institute of Science, Bangalore, India and were maintained in the animal house of the Institute. The animals were housed in polypropylene cages and provided standard pellet diet (Agro Corporation Pvt. Ltd., Bangalore, KA, India) and water *ad libitum*. The standard pellet diet given to the mice contains 21% protein, 6.7% lipids, 3% crude fibre, 8% ash, 1.5% calcium, 0.9% phosphorus, 3.4% glucose, 2% vitamin and 57% nitrogen-free extract (carbohydrates) obtained from Provomi Animal Nutrition India Pvt.Ltd. Bangalore, India. The mice were maintained in optimum temperature and humidity with a 12 h light/dark cycle.

### Administration of Elvitegravir in mice

Out of 31 Balb/c mice, 16 were fed with Elvitegravir (30 mg/kg body weight) using oral gavage. The concentration of compound fed to mice was equivalent to a single dose of 150 mg/day given to a human patient. Group 1, that served as the untreated (normal, *n*=11) control were treated with an equivalent amount of vehicle control (5% methylcellulose). Group 2 mice were treated with Cobicistat alone (30 mg/Kg body weight, *n*=4) and the Group 3 mice were treated with Elviteravir and Cobicistat (30 mg/Kg each; *n*=16). Treatment with the drug started after 2 weeks of mice birth, i.e., from 15th day and compound was fed to the mice every day for 8 days. On the 9th day after the starting of treatment with the Elvitegravir, mice were sacrificed; bone marrow cells were collected and processed to study the effect of Elvitegravir on different cell population of B and T cells. Each experiment was repeated three independent times.

### Flow cytometric analysis of mice lymphocytes upon treatment with Elvitegravir

To study the effect of Elvitegravir on B cell population, mice were dissected; femurs and tibias from both the hind legs were collected. Bones were flushed with PBS to remove the bone marrow cells. Gentle RBC lysis was given using distilled water for 4–5 s. Bone marrow cells were washed with PBS and were blocked in 2% BSA, 2% FBS in 1 × PBS and probed with appropriate cell surface markers, anti-CD25-PE Cy7 and anti-CD45-APC for B cells.

To further assess the percentage of B and T cells in the lymphocyte population of bone marrow cells, anti-CD19-APC and anti-CD25-PE Cy7 were used as B cell surface markers and anti-CD3-FITC, anti-CD4-PE, and anti-CD8-APC Cy7 were used for T cells. Cells were washed with PBS containing 1% BSA and analysed by flow cytometry (BD Biosciences FACS Verse). Via forward and side scatter, lymphocytes were gated and dot plots were plotted with BD FACS DIVA software version 6.1.3 and analysed.

### Statistical analyses

The significance for inhibition of RAG-mediated binding, cleavage and hairpin formation was determined statistically. Each experiment was repeated a minimum of three times, and significance was calculated in GraphPad Prism 5 using one-way ANOVA and Dunnett's statistical significance test. In all cases, the significance of the difference between control and each concentration of inhibitor used is plotted. *P*-value is represented as: *P<0.05, **P<0.005, ***P<0.0001.
